# Knowledge, attitudes, and behavioural risk factors regarding zoonotic infections among bushmeat hunters and traders in Nsukka, southeast Nigeria

**DOI:** 10.4178/epih.e2018025

**Published:** 2018-06-16

**Authors:** Kingsley Uchenna Ozioko, Chris Ikem Okoye, Rose Nduka Obiezue, Raymond Awudu Agbu

**Affiliations:** 1 Parasitology and Public Health Research Unit, Department of Zoology and Environmental Biology, University of Nigeria, Nsukka, Nigeria; 2 Central Laboratory Unit, Federal University Wukari, Wukari, Nigeria

**Keywords:** Knowledge, Attitude, Risk, Zoonotic, Wildlife, Pathogen

## Abstract

**OBJECTIVES:**

In light of the dramatic spread of Ebola virus in some parts of Africa and the 2014 outbreak in Nigeria, a study was conducted to evaluate bushmeat dealers’ knowledge and attitudes about zoonotic infections and the risk of transmission to humans.

**METHODS:**

A cross-sectional survey was conducted in a community in Nsukka, southeast Nigeria. Hunters (n=34) and bushmeat traders (n=42) were interviewed. A semi-structured questionnaire was used to generate the data. The Fisher exact test was used to evaluate the significance of differences between these groups.

**RESULTS:**

Only 11.8% of the hunters, as compared to 35.7% of the traders, had no knowledge of possible causes of zoonotic infections (p<0.05). However, 64.7% of the hunters, compared to 38.1% of the traders, were ignorant regarding the responsibility of public health personnel and veterinarians (p<0.05), and 76.5% of the hunters compared to 42.9% of the traders were ignorant regarding the existence of zoonoses in Nigeria (p<0.05). A statistically significant difference was also found between these groups regarding the risk of contracting an infection from ectoparasites (p<0.05). The attitudes of respondents towards zoonotic diseases did not differ significantly between the groups.

**CONCLUSIONS:**

The level of awareness about zoonotic diseases was low in this area, underscoring the need for interventions.

## INTRODUCTION

Wildlife serves as food for males and as a reservoir host of pathogens that cause disease in males. Human contacts with a wide range of wildlife tissues and fluids, either directly or indirectly through arthropod bites during hunting and processing of bushmeat, expose humans to a risk of zoonoses [1]. Certain individuals with occupational exposure, such as traders (chop bar owners, bushmeat vendors), or individuals who participate in outdoor recreational activities, such as hunters, are at a higher risk of contracting and spreading infections from bushmeat. The percentage of wild animals that are carriers for zoonotic diseases is increasing, resulting in growing concerns for human safety and control [1]. Awareness is the most important strategy for preventing occupationally acquired infectious diseases. Thus, knowledge and awareness about the risk of zoonotic diseases is a prerequisite for effective disease control [2]. Zoonotic infections are among the most common on earth, accounting for more than 60% of all human infectious diseases [3]. Most emerging zoonotic diseases are of viral origin and are transmitted by vectors [4], which for the most part are wildlife [5,6]. Some of the most notorious disease outbreaks of all time were the result of zoonotic pathogens. Perhaps the most widely known of these pathogens is the Ebola virus. This disease has led to major outbreaks in some parts of Africa, and may have killed millions of people throughout several centuries. The route of transmission has been linked to bushmeat, and unusual behaviours exhibited by the inhabitants of Nsukka during the outbreak in Nigeria were alarming. There was an obvious reduction in the demand for bushmeat consumption in the area, to the point that the Association of Bushmeat Traders and Hunters had to organise an event highlighting public consumption of their wares in public, in an effort to boost demand. Nigeria was declared to be free of Ebola virus disease by the World Health Organisation on October 20, 2014, but the federal government has put in place an emergency programme to monitor all border activity to keep Nigeria safe following the 2018 outbreak in the Democratic Republic of the Congo [7]. Therefore, assessing the knowledge, attitudes, and behavioural risk factors associated with the prevention and control of zoonotic diseases in Nigeria is crucial in order to identify gaps that may encourage the spread of any outbreak of similar diseases in the future. In Nsukka, wildlife has been associated with several important zoonotic pathogens [7]. Currently, inadequate data exist on the knowledge, attitude, and behavioural risk factors for zoonotic diseases in Nsukka generally and for bushmeat-borne zoonotic diseases in particular. Therefore, the objective of this study was to assess the level of knowledge, attitudes, and behavioural risk factors regarding zoonotic infections among the bushmeat dealers in the study area. The intention is that the baseline information generated will facilitate the development of effective joint veterinary– medical policies and guidelines for controlling emerging zoonotic diseases in the area. Collecting information and assessing safe work practices and attitudes among at-risk populations can provide a suitable format to evaluate existing programs and to identify effective strategies for behavioural changes.

## MATERIALS AND METHODS

### Study area and population

This study was carried out between October 2016 and February 2017 among bushmeat dealers based in the Nsukka Senatorial Zone of Enugu State, Nigeria. Nsukka is located in the northern part of Enugu State, between longitude 7°08’ and 7°20’ east and latitude 6°46’ and 6°49’ north [8]. It is made up of 7 densely populated Local Government Areas: Igbo-Etiti, Uzo-Uwani, Isi-Uzo, Nsukka, Udenu, Igbo-Eze North, and Igbo-Eze South. Its population accounts for over 49% of Enugu State. As of 2007, the Nsukka Cultural Zone had an estimated population of 1,377,001 [8]. The town of Nsukka is known as the location of the first full-fledged indigenous university in Nigeria (University of Nigeria, Nsukka). Bushmeat is transported from forests and grassland areas, where hunting mainly takes place, to markets within local communities or to larger markets in urban centres, where it is then sold to bushmeat traders who buy them directly from hunters (the source of the supply).

### Sample size

No sampling was done. All registered full-time hunters and traders during the given period were eligible to take part in the study. The total number of subjects was 76.

### Study design

The study was designed to cover all registered hunters in the area who hunted and transported bushmeat to markets and all registered traders who bought them directly from the hunters, because doing so was considered feasible. A compilation of names was first produced through the local chairmen of bushmeat association. The list was used to contact them for the data collection process, which encountered some delays, as some had no mobile phones. Given the buy-in from union chairmen, all dealers were willing to participate in the study. The questionnaire was divided into 4 major areas. The category of demographics included 3 questions that provided vital information covering a wide range of socio-demographic areas. Basic knowledge about zoonotic infections was assessed through 5 questions that were related to different aspects of zoonoses, ranging from basic and general information to knowledge about transmission and prevention. The section on attitudes towards zoonoses contained 4 yes/no statements measuring the attitude of respondents towards different aspects of zoonoses. The category of behavioural risk factors for zoonotic infections had 4 questions covering basic sources of zoonotic links.

### Data collection

A semi-structured questionnaire was used to obtain data from the participants. The focus of the questionnaire was on aspects of registered full-time hunters and traders’ knowledge that were considered important for the identification and perception of zoonoses. A total of 42 bushmeat traders and 34 bushmeat hunters responded to an interviewer-guided questionnaire, which probed demographic data and information on knowledge, attitudes, and risk factors related to zoonoses, with a particular emphasis on bushmeat-borne zoonoses. It was designed to be completed within 20 minutes for an average respondent. All technical terms in the questionnaire were translated into the Igbo language and explained by the interviewer. Confidentiality was assured to each participant, and demographic data of the study participants, including sex, marital status, and educational status, were collected and presented in a tabular format.

### Data analysis

The collected data were processed using SPSS version 20 (IBM Corp., Armonk, NY, USA). The Fisher exact test was used to evaluate the significance differences, with p≤ 0.05 considered to indicate significant differences. Frequency and percentage were computed for all variables.

### Ethical statement

Ethical approval was not required in this study, although authorization was sought and received from the chairman of the Association of Hunters and Bushmeat Traders. At an individual level, verbal consent was received from each participant before data collection.

## RESULTS

### Demographic characteristics

Of the 76 participants, more were male (65.8%) than female (34.2%) (Table 1). As reported by the respondents, 81.6% were married and 18.4% were single. In terms of educational status, 78.9% of the respondents had a secondary education, 18.4% had only a primary education, and 1 hunter (2.6%) had a tertiary education.

### Knowledge of respondents (hunters and traders) about zoonotic diseases

In order to gain an understanding of what participants knew, understood, and believed regarding zoonotic diseases and health, participants were asked to supply information based on their level of awareness (Table 2). Very few participants were aware of the existence of zoonotic infections that could be transmitted by handling, processing, or eating bushmeat. Regarding knowledge of the causes of zoonotic infections, 11.8% of the hunters had no knowledge of the possible cause of such infections, compared to 35.7% of the traders, which was a significant difference between the groups (p< 0.05). Approximately half of the hunters (73.5%) and the traders (52.4%) were not aware of how to reduce the likelihood of contracting work-related infections. A significant difference was found between hunters (64.7%) and traders (38.1%) in terms of views regarding the responsibility of public health personnel and veterinarians for providing information about zoonotic infections (p< 0.05). A statistically significant difference was also found between the groups in terms of awareness of the existence of zoonoses in wild animals in Nigeria, as 76.5% of hunters thought that there were none, compared to 42.9% of traders (p< 0.05).

### Attitudes of respondents (hunters and traders) towards zoonotic diseases

A majority of respondents had negative attitudes towards zoonotic infections (Table 3). However, 52.9% of the hunters and 28.6% of the traders expressed no concern that they might acquire an infection from bushmeat. Regarding wearing protective gear during contact with animals, the overwhelming majority of respondents (85.3 and 88.1% of hunters and traders, respectively) considered doing so to be unnecessary. Correspondingly, a high percentage of both the hunters (91.2%) and the traders (76.2%) valued bushmeat more than their health. The vast majority of respondents (94.1% of the hunters and 90.5% of the traders) did not seek medical consultations regarding the possibility of a zoonotic disease when sick. Attitudes towards zoonotic diseases did not differ significantly between the groups.

### Behavioural risk factors for zoonotic infections (hunters and traders)

Table 4 presents the distribution of responses regarding behavioural risk factors for zoonotic diseases. Behavioural risk factors for zoonosis varied significantly between the groups (p< 0.05). A high percentage of traders and hunters had never in their life contracted an infection from bushmeat (91.2 and 90.5%, respectively). Furthermore, 88.2% of the hunters stated that they had been bitten by wildlife used for bushmeat within the last 12 months, while 95.2% of the traders said that they had not (p< 0.001). Additionally, 85.3 and 38.1% of the hunters and traders, respectively, reported sharing their house with animals or processing animals at home regularly (at least weekly), which was a significant difference between the groups (p< 0.001). Only a few of the participants were aware of the risk of direct transmission via contact with the placenta or skin-to-skin contact with animals when processing or sharing their house with a wild animal.

## DISCUSSION

Up-to-date knowledge, attitudes, and behaviours regarding zoonotic diseases among high-risk groups are vital for the formulation and effective implementation of appropriate disease prevention and control strategies in any given area [9]. The results obtained from this study demonstrated that male respondents represented about 65.8% of those who were involved in processing and hunting bushmeat; this shows that it was entirely male occupation in the study area, meaning that men were at the highest risk for bushmeat-borne zoonotic infections. Participants’ literacy level seemed to be sufficiently high, as 78.9% of the respondents had attended secondary education, while 2.6% reported a tertiary-level education. This could also explain why there was insufficient knowledge about zoonoses. Sixty-two (81.6%) of the respondents were married, which implies that bushmeat hunting and processing were not mostly carried out by youth in the study area; hence, control and preventive measures for zoonosis exposure should also be targeted at this more mature group. The study identified that the overall level of awareness and knowledge about zoonoses among bushmeat suppliers was low, which is in line with a study done in Arusha and Tanga, Tanzania. The risk of contracting work-related diseases was high among the participants, in accordance with a previous study [10] that reported such a risk in 90.0% of the participants. Hunters were at a significantly higher risk than traders (p < 0.05), so awareness of preventive measures should be targeted at this group as well. The results of this study showed that the majority of participants who had ever heard of zoonoses had the false belief that such diseases cannot be transmitted from bushmeat to humans in Nigeria. This incorrect belief may spread to other people within the country, ultimately contributing to poor disease control [11]. The low level of zoonotic disease awareness observed in the participants was perhaps not surprising. Approximately half of the participants knew that public health personnel and veterinarians are responsible for reporting zoonoses. This suggests that the majority of veterinarians and physicians do not regularly discuss zoonotic disease risks with bushmeat dealers [12]. This study indicated that a good number of participants valued their animals more than their health, which agrees with the previous finding [13] that the quality of bushmeat appears to be a reason why many consumers overlook the potential for disease or infection. The majority of the participants did not agree that it is the responsibility of public health personnel and veterinarians to provide information about bushmeat-associated infections or precautions to reduce the risk of these infections, which was not surprising. Although some participants expressed concerns about contracting a work-related zoonosis, exposure to infection occurred due to lack of awareness. Bushmeat hunters and traders were, for the most part, not concerned about bushmeat-associated zoonoses and were comfortable with their level of knowledge and methods to reduce zoonotic disease risk. As individuals who are not concerned, and are comfortable with their knowledge base, may be unlikely to seek additional knowledge from available resources [14], active methods may be required to improve awareness of bushmeat-associated zoonoses. A high percentage of the participants stated that they had never in their life contracted an infection from bushmeat. However, most of them reported not seeking a medical consultation regarding the possibility of a zoonotic disease when sick; instead, they sought out alternative medicines. A very large percentage of the participants stated that someone in their household had physical contact with animals outside the home on a weekly basis, which is another transmission pathway and a possible zoonotic link within the human population. Some of the respondents had good knowledge and positive attitudes about zoonotic infections, but lacked knowledge about the causative agent and the mode of transmission. Since their close associations with animals make hunters and bushmeat traders a high-risk group for contacting wildlife-associated zoonotic diseases, it is advisable that they undergo periodic health check-ups for the early diagnosis and treatment of zoonoses [15].

In conclusion, the level of knowledge, attitudes, and behavioural risks related to bushmeat-borne zoonoses among bushmeat dealers and hunters in Nsukka, southeast Nigeria were investigated in this study. Weaknesses were identified in knowledge and attitudes, as well as in practices that are likely to expose them to an increased risk of contracting zoonoses, as the respondents reported being unlikely to take proper precautions. Therefore, it is necessary for health personnel to educate the inhabitants of this area on the risk of zoonoses in general and to launch appropriate interventions. The establishment of bushmeat information centres in villages or at the ward level might also be useful for enhancing knowledge and skills and improving awareness and efficient control of the spread of zoonotic infections. This study provided a thorough comparison of the differences between hunters and traders in terms of knowledge, attitudes, and behavioural risk factors regarding zoonotic infections. This will help direct policymakers in providing distinctive resources corresponding to the needs or challenges of each group. It should be kept in mind, however, that participants may report answers that do not reflect their norms or state that they have common beliefs, which may impair the validity of the conclusions. Among demographic factors, age is a very important factor regarding knowledge, attitudes, and practices, but it was not separately analysed in this study since all registered hunters and traders were adults. Future research may additionally examine bushmeat consumers.

## Figures and Tables

**Table 1.** Demographic characteristics of the subjects

Characteristics	Hunters (n=34)	Traders (n=42)	Total (n=76)
Sex			
Male	34	16	50 (65.8)
Female	-	26	26 (34.2)
Educational status			
Primary level	4	10	14 (18.4)
Secondary level	28	32	60 (78.9)
Tertiary level	2	-	2 (2.6)
Marital status			
Married	22	40	62 (81.6)
Single	12	2	14 (18.4)

Values are presented as number or number (%).

**Table 2.** Respondents’ knowledge of zoonotic diseases

Variables	Hunters (n=34)	Traders (n=42)	p-value
Knowledge regarding the existence of zoonoses			
Yes	5 (14.7)	4 (9.5)	0.50
No	29 (85.3)	38 (90.5)	
Knowledge that zoonotic diseases are caused by harmful germs			
Yes	30 (88.2)	27 (64.3)	0.02
No	4 (11.8)	15 (35.7)	
Knowledge of ways to reduce zoonotic infections			
Yes	9 (26.5)	20 (47.6)	0.10
No	25 (73.5)	22 (52.4)	
Knowledge that public health personnel and veterinarians are responsible for reporting zoonoses			
Yes	12 (35.3)	26 (61.9)	0.04
No	22 (64.7)	16 (38.1)	
Knowledge that one can be infected here in Nigeria			
Yes	8 (23.5)	24 (57.1)	0.005
No	26 (76.5)	18 (42.9)	

Values are presented as number (%).

**Table 3.** Respondents’ attitudes towards zoonotic diseases

Variables	Hunters (n=34)	Traders (n=42)	p-value
Concerned about contracting a work-related zoonosis			
Yes	16 (47.1)	30 (71.4)	0.36
No	18 (52.9)	12 (28.6)	
Concerned about using protective measures			
Yes	5 (14.7)	5 (11.9)	0.74
No	29 (85.3)	37 (88.1)	
Agree that benefits of bushmeat consumption do not outweigh any health risks			
Yes	3 (8.8)	10 (23.8)	0.13
No	31 (91.2)	32 (76.2)	
Seek a medical consultation when needed			
Yes	2 (5.9)	4 (9.5)	1.00
No	32 (94.1)	38 (90.5)	

Values are presented as number (%).

**Table 4.** Behavioural risk factors for zoonotic infections

Variables	Hunters (n=34)	Traders (n=42)	p-value
Have previously contracted an infection from bushmeat			
Yes	3 (8.8)	4 (9.5)	1.00
No	31 (91.2)	38 (90.5)	
Have been bitten by wildlife used for bushmeat within 12 mo			
Yes	30 (88.2)	2 (4.8)	<0.001
No	4 (11.8)	40 (95.2)	
Have been bitten by ectoparasites			
Yes	26 (76.5)	35 (83.3)	0.56
No	8 (23.5)	7 (16.7)	
Processes bushmeat at home or shares house with wild animals			
Yes	29 (85.3)	16 (38.1)	<0.001
No	5 (14.7)	26 (61.9)	

Values are presented as number (%).
